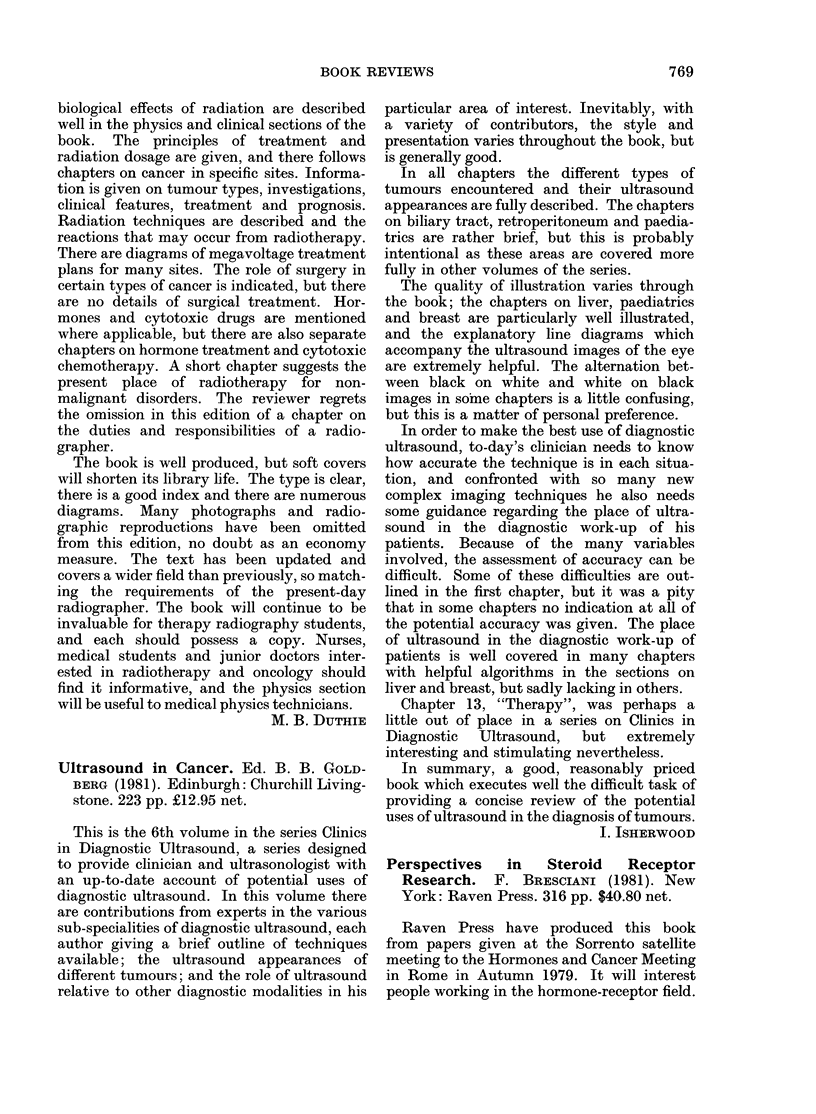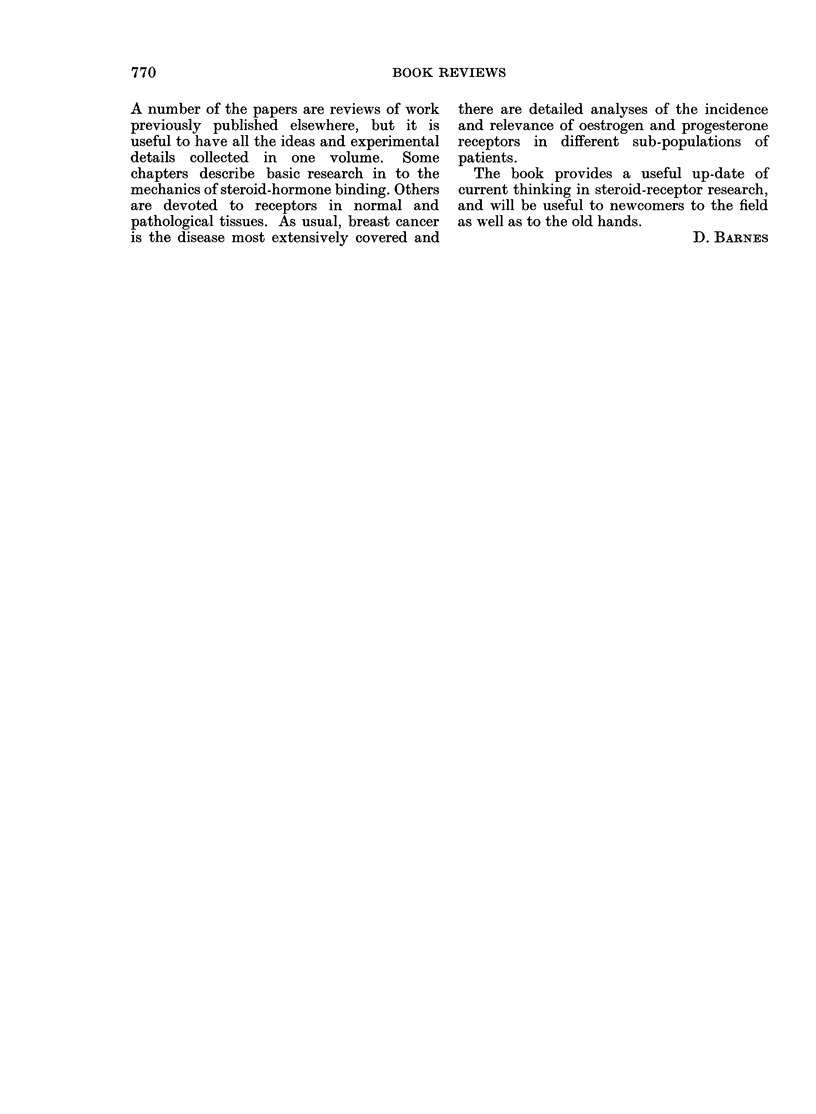# Perspectives in Steroid Receptor Research

**Published:** 1981-11

**Authors:** D. Barnes


**Perspectives in Steroid Receptor Research.** F. BRESCIANI (1981). New York: Raven Press. 316 pp. $40.80 net.

Raven Press have produced this book from papers given at the Sorrento satellite meeting to the Hormones and Cancer Meeting in Rome in Autumn 1979. It will interest people working in the hormone-receptor field.

A number of the papers are reviews of work previously published elsewhere, but it is useful to have all the ideas and experimental details collected in one volume. Some chapters describe basic research in to the mechanics of steroid-hormone binding. Others are devoted to receptors in normal and pathological tissues. As usual, breast cancer is the disease most extensively covered and there are detailed analyses of the incidence and relevance of oestrogen and progesterone receptors in different sub-populations of patients.

The book provides a useful up-date of current thinking in steroid-receptor research, and will be useful to newcomers to the field as well as to the old hands.

D. Barnes